# Using gene-environment interactions to explore pathways for colorectal cancer risk

**DOI:** 10.1016/j.ebiom.2025.105964

**Published:** 2025-10-11

**Authors:** Emmanouil Bouras, Ren Yu, Andre E. Kim, Georgios Markozannes, Neil Murphy, Demetrius Albanes, Laura N. Anderson, Elizabeth L. Barry, Sonja I. Berndt, D. Timothy Bishop, Hermann Brenner, Andrea Burnett-Hartman, Peter T. Campbell, Robert Carreras-Torres, Andrew T. Chan, Iona Cheng, Matthew A. Devall, Virginia Diez-Obrero, Niki Dimou, David A. Drew, Stephen B. Gruber, Andrea Gsur, Michael Hoffmeister, Li Hsu, Jeroen R. Huyghe, Eric Kawaguchi, Temitope O. Keku, Anshul Kundaje, Sébastien Küry, Loïc Le Marchand, Juan Pablo Lewinger, Li Li, Brigid M. Lynch, Victor Moreno, John L. Morrison, Christina C. Newton, Mireia Obón-Santacana, Julie R. Palmer, Nikos Papadimitriou, Andrew J. Pellatt, Anita R. Peoples, Paul D.P. Pharoah, Elizabeth A. Platz, Conghui Qu, Edward Ruiz-Narvaez, Joel Sanchez Mendez, Robert E. Schoen, Mariana C. Stern, Claire E. Thomas, Yu Tian, Caroline Y. Um, Kala Visvanathan, Pavel Vodicka, Veronika Vymetalkova, Emily White, Alicja Wolk, Michael O. Woods, Anna H. Wu, Marc J. Gunter, W. James Gauderman, Ulrike Peters, Marina Evangelou, Konstantinos K. Tsilidis

**Affiliations:** a Department of Epidemiology and Biostatistics, School of Public Health—Faculty of Medicine, Imperial College London, London, UK; b Department of Hygiene and Epidemiology, University of Ioannina School of Medicine, Ioannina, Greece; c Department of Mathematics, Faculty of Natural Sciences, Imperial College London, London, UK; d Division of Biostatistics, Department of Population and Public Health Sciences, Keck School of Medicine, University of Southern California, Los Angeles, CA, USA; e Nutrition and Metabolism Branch, International Agency for Research on Cancer, Lyon, France; f Division of Cancer Epidemiology and Genetics, National Cancer Institute, National Institutes of Health, Bethesda, MD, USA; g Department of Health Research Methods, Evidence, and Impact, McMaster University, Hamilton, ON, Canada; h Department of Epidemiology, Geisel School of Medicine at Dartmouth, Hanover, NH, USA; i Leeds Institute of Cancer and Pathology, University of Leeds, Leeds, UK; j Division of Clinical Epidemiology and Aging Research, German Cancer Research Center (DKFZ), Heidelberg, Germany; k Division of Preventive Oncology, German Cancer Research Center (DKFZ) and National Center for Tumor Diseases (NCT), Heidelberg, Germany; l German Cancer Consortium (DKTK), German Cancer Research Center (DKFZ), Heidelberg, Germany; m Institute for Health Research, Kaiser Permanente Colorado, Denver, CO, USA; n Department of Epidemiology and Population Health, Albert Einstein College of Medicine, Bronx, NY, USA; o Colorectal Cancer Group, ONCOBELL Program, Bellvitge Biomedical Research Institute (IDIBELL), L'Hospitalet de Llobregat, 08908, Barcelona, Spain; p Digestive Diseases and Microbiota Group, Girona Biomedical Research Institute (IDIBGI), Salt, 17190, Girona, Spain; q Broad Institute of Harvard and MIT, Cambridge, MA, USA; r Channing Division of Network Medicine, Brigham and Women's Hospital and Harvard Medical School, Boston, MA, USA; s Clinical and Translational Epidemiology Unit, Massachusetts General Hospital and Harvard Medical School, Boston, MA, USA; t Division of Gastroenterology, Massachusetts General Hospital and Harvard Medical School, Boston, MA, USA; u Department of Epidemiology and Biostatistics, University of California-San Francisco, San Francisco, CA, USA; v Center for Public Health Genomics, Department of Public Health Sciences, University of Virginia, Charlottesville, VA, USA; w Department of Public Health Sciences, Center for Public Health Genomics, Charlottesville, VA, USA; x Unit of Biomarkers and Susceptibility (UBS), Oncology Data Analytics Program (ODAP), Catalan Institute of Oncology (ICO), Barcelona, 08908, Spain; y Consortium for Biomedical Research in Epidemiology and Public Health (CIBERESP), 28029, Madrid, Spain; z Department of Clinical Sciences, Faculty of Medicine, University of Barcelona, Barcelona, 08908, Spain; aa Department of Medical Oncology & Therapeutics Research and Center for Precision Medicine, City of Hope National Medical Center, Duarte, CA, USA; ab Center for Cancer Research, Medical University of Vienna, Vienna, Austria; ac Public Health Sciences Division, Fred Hutchinson Cancer Center, Seattle, WA, USA; ad Department of Biostatistics, University of Washington, Seattle, WA, USA; ae Center for Gastrointestinal Biology and Disease, University of North Carolina, Chapel Hill, NC, USA; af Department of Computer Science, Stanford University, Stanford, CA, USA; ag Department of Genetics, Stanford University, Stanford, CA, USA; ah Service de Génétique Médicale, Centre Hospitalier Universitaire (CHU) Nantes, Nantes, France; ai Epidemiology Program, University of Hawaii Cancer Center, Honolulu, HI, USA; aj Department of Family Medicine, University of Virginia, Charlottesville, VA, USA; ak Cancer Epidemiology Division, Cancer Council Victoria, Melbourne, Victoria, Australia; al Centre for Epidemiology and Biostatistics, Melbourne School of Population and Global Health, The University of Melbourne, Melbourne, Victoria, Australia; am Department of Population Science, American Cancer Society, Atlanta, GA, USA; an Slone Epidemiology Center, at Boston University, Boston, MA, USA; ao Intermountain Health, Salt Lake City, UT, USA; ap Department of Public Health and Primary Care, University of Cambridge, Cambridge, UK; aq Department of Epidemiology, Johns Hopkins Bloomberg School of Public Health, Baltimore, MD, USA; ar Department of Nutritional Sciences, University of Michigan School of Public Health, Ann Arbor, MI, USA; as Department of Population and Public Health Sciences, Keck School of Medicine, University of Southern California, Los Angeles, CA, USA; at Departments of Medicine and Epidemiology, University of Pittsburgh, Pittsburgh, PA, USA; au Division of Cancer Epidemiology, German Cancer Research Center (DKFZ), Heidelberg, Germany; av School of Public Health, Capital Medical University, Beijing, China; aw Department of Molecular Biology of Cancer, Institute of Experimental Medicine of the Czech Academy of Sciences, Prague, Czech Republic; ax Faculty of Medicine and Biomedical Center in Pilsen, Charles University, Pilsen, Czech Republic; ay Institute of Biology and Medical Genetics, First Faculty of Medicine, Charles University, Prague, Czech Republic; az Department of Epidemiology, University of Washington School of Public Health, Seattle, WA, USA; ba Institute of Environmental Medicine, Karolinska Institutet, Stockholm, Sweden; bb Memorial University of Newfoundland, Discipline of Genetics, St. John's, Canada

**Keywords:** Colorectal cancer, Gene-environment interactions, Molecular pathways, Mechanisms, Pathway analysis

## Abstract

**Background:**

Colorectal cancer (CRC) is a significant public health concern, highlighting the critical need for identifying novel intervention targets for its prevention.

**Methods:**

We conducted genome-wide interaction analyses for 15 exposures with established or putative CRC risk [body mass index (BMI), height, physical activity, smoking, type 2 diabetes, use of menopausal hormone therapy, non-steroidal anti-inflammatory drugs, and intake of alcohol, calcium, fibre, folate, fruits, processed meat, red meat, and vegetables], and used interaction estimates to explore pathways and genes underlying CRC risk. The adaptive combination of Bayes Factors (ADABF), and over-representation analysis (ORA) were used for pathway analyses, and findings were further investigated using publicly available resources [hallmarks of cancer, Open Targets Platform (OTP)].

**Findings:**

A total of 1973 pathways using ADABF, and 840 pathways using ORA, out of the 2950 analysed, were enriched (*P* < 0.05) for at least one exposure, as well as 1227 genes within the enriched pathways. Data were available for 811/1227 coding genes in the OTP, 241 of which were supported by strong relative abundance of prior evidence (overall OTP score > 0.05). Fifty percent of the genes (617/1227) mapped to at least one hallmark of cancer, most of which (388/617) pertained to the Sustaining Proliferative Signalling hallmark. Our findings reflect previously established pathways for CRC risk and highlight the emerging importance of several less studied genes. Common pathways were found for several combinations of exposures, potentially suggesting common underlying mechanisms.

**Interpretation:**

The results of the present analysis provide a basis for further functional research. If confirmed, they may help elucidate the etiological associations between risk factors and CRC risk and ultimately inform personalized prevention strategies.

**Funding:**

This study was funded by *Cancer Research UK* (CRUK; *grant number:**PPRCPJT∖100005*) and World Cancer Research Fund International (WCRF; *IIG_FULL_2020_022*). Funding for grant IIG_FULL_2020_022 was obtained from Wereld Kanker Onderzoek Fonds (WKOF) as part of the World Cancer Research Fund International grant programme. Full funding details for the individual consortia are provided in the acknowledgements.

**Research in Context**

**Evidence before this study**

Gene-environment interaction analyses have identified several associations and mechanisms related to colorectal cancer (CRC) risk. However, they typically focus on a limited set of genetic loci that remain statistically significant after correction for multiple comparisons, leaving potentially suggestive associations underexploited.

**Added value of this study**

We derived gene-environment interaction estimates for 15 CRC risk factors (e.g., BMI, smoking, diet, medications) across genes mapped to 2950 biological pathways, and conducted pathway analyses, to highlight genes and pathways for CRC risk. The relevance of the identified genes and pathways to CRC was further assessed using publicly available resources. Our findings support the involvement of several genes and pathways, many of which have been relatively understudied in the context of CRC. Notably, we identified pathways and genes that were enriched across multiple exposures, suggesting the presence of shared underlying mechanisms.

**Implications of all the available evidence**

The results of the present analysis provide a basis for further functional research. If confirmed, they may help elucidate the etiological associations between risk factors and CRC risk and ultimately inform personalized prevention strategies.

## Introduction

Colorectal cancer (CRC) is the third most common type of cancer globally, and the second cause of cancer death, with an estimate of approximately two million incident cases and one million deaths in 2020.^1^ There has been an upward trend over the past decades, with projections of incidence cases estimated to increase by more than 60% by 2040.^2^ Such an increase is largely driven by growth of the older population, but also reflects changes in the prevalence of major CRC risk factors, such as smoking, adiposity, and physical inactivity,^3^ especially among younger adults in prescreening ages (<50).^4^

Investigating the interactions between genome-wide variants and relevant exposures for risk of CRC is useful in identifying novel risk loci, but also offers insights into the biological pathways that are implicated in the development of CRC.^5^ Such gene-environment interaction analyses have yielded several associations for CRC risk (and potentially mechanisms), such as in loci 6p21.33 [Activating Transcription Factor 6 Beta (*ATF6B*)] and 3p12.1 [Cell Adhesion Molecule 2 (*CADM2*)] as interaction loci with smoking, 10q24.2 [Cytochrome C Oxidase Assembly Homolog COX15 (*COX15*)] with alcohol consumption, 12p13.1 [Glutamate Ionotropic Receptor NMDA Type Subunit 2B (*GRIN2B*)] and 6q22.1 [Discoidin, CUB And LCCL Domain Containing 1 (*DCBLD1*)] with menopausal hormone therapy (MHT) use, 8q24.11 [Solute Carrier Family 30 Member 8 (*SLC30A8*)] with diabetes, 7q31.1 [Solute Carrier Family 26 Member 3 (*SLC26A3*)] with fibre, 1p31.1 [Neuronal Growth Regulator 1 (*NEGR1*)] with fruit consumption, 8q24.13 [Hyaluronan Synthase 2 (*HAS2*)] and 18q21.1 [SMAD Family Member 7 (*SMAD7*)] with red meat consumption, 3p25.2 [Synapsin II (*SYN2*)/tissue inhibitor of metalloproteinase 4 (*TIMP4*)] with folate, 15q13.3 [Formin 1/Gremlin 1 (FMN1/GREM1)] with body mass index (BMI).^6^, ^7^, ^8^, ^9^, ^10^, ^11^, ^12^, ^13^ These high-throughput analyses entail a wide genetic coverage but typically focus on the limited set of genetic loci that remain statistically significant after correction for multiple comparisons, leaving a wealth of information (including potentially suggestive associations) inevitably underexploited. A holistic investigation of those associations may shed light on novel pathways for CRC risk and identify targets for CRC prevention, which is of critical importance due to the rising incidence.

In this study, we aimed to investigate biological pathways linked to CRC development by conducting genetic pathway-environment interaction analyses. Using estimates from genome-wide interaction studies (GWIS) of 15 exposures with established or putative CRC risk, we examined associated and over-represented genes and pathways in a pooled dataset of 36 studies including approximately 85,000 participants of European ancestry.

## Methods

**Fig. 1:**
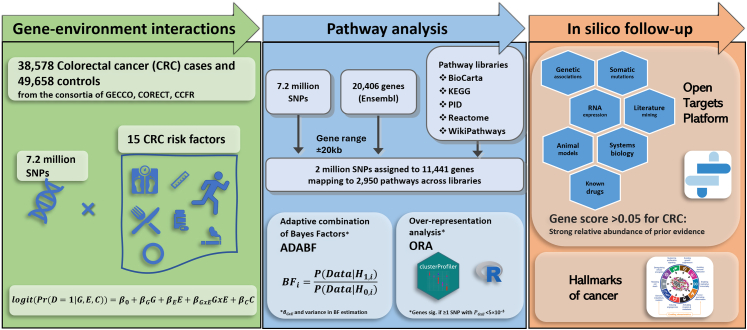
Project overview. ADABF: adaptive combination of Bayes factors; BF: Bayes factors; CCFR: Colon Cancer Family Registry Cohort; CRC: Colorectal cancer; GECCO: Genetics and Epidemiology of Colorectal Cancer Consortium; KEGG: Kyoto Encyclopedia of Genes and Genomes; ORA: Over-representation analysis; PID: Pathway Interaction Database; SNPs: Single nucleotide polymorphisms.

The overview of the project is presented in Fig. 1.

### Study participants

Details on the study design and the GWIS are described elsewhere.^6^, ^7^, ^8^, ^9^, ^10^, ^11^, ^12^, ^13^ In brief, we included data from a total of 36,415 CRC cases and 48,451 controls from 36 studies contributing to three consortia—the multi-centred Colon Cancer Family Registry Cohort (CCFRC), the Genetics and Epidemiology of Colorectal Cancer Consortium (GECCO), and the Colorectal Transdisciplinary Study (CORECT) (S.Table 1). Cases were confirmed by medical records, pathological reports, or death-certificate information, and controls were matched on age, sex, and enrolment date/trial group, when applicable. All participants gave written informed consent and studies were approved by their respective Institutional Review Boards/ethnic committees. This investigation was restricted to European-ancestry participants.

### Exposures

Information on exposures and other variables of interest, such as demographics and environmental risk factors, was obtained using structured questionnaires and/or interviews, and a multi-step data-harmonization procedure was carried out at the GECCO coordinating centre (Fred Hutchinson Cancer Center), to reconcile each study's protocols and data-collection instruments.^14^ We used 15 exposures (27 separate variables), namely alcohol, calcium, fibre, folate, fruit, processed meat, red meat, and vegetable intake, BMI, height, physical activity, smoking, type 2 diabetes (T2D), MHT, non-steroidal anti-inflammatory drugs (NSAIDs), and several sub-categories [e.g., smoking was assessed as a categorical variable (ever vs. never smoker) and as a continuous variable (pack-years of smoking)] (S.Table 2). These exposures were prioritized for analysis based on prior evidence of their relevance to colorectal cancer and have been systematically harmonized across participating studies within the GECCO consortium. Exposure specific descriptives and sample sizes are presented in S.Table 3.

### Genotyping, quality assurance/control and imputation

Details on quality control and genotyping have been previously described.^15^^,^^16^ Briefly, genotyping call rates (<97%), heterozygosity, duplicates or next of kin individuals, and discrepancies between self-reported and genotypic sex, were used as criteria to exclude study participants, that were further limited to only European ancestry, as determined by self-reported race and principal components clustering with 1000 Genomes EUR super-populations.^17^ Genetic markers were excluded based on missing call rates (>2–5%), departures from Hardy–Weinberg Equilibrium (HWE) (*P*-value <1 × 10^−4^), and discordant genotype calls within duplicate samples. Genotypes were imputed to the Haplotype Reference Consortium (HRC version r1.1) using the University of Michigan Imputation Server and filtered out imputed single nucleotide polymorphisms (SNPs) based on imputation accuracy (R^2^ > 0.8) and minor allele frequency (MAF) > 1%, leaving a total of more than 7.2 million SNPs that were used in subsequent interaction analyses. Principal component analysis (PCA) for population stratification assessment was performed using PLINK1.9.^15^^,^^16^

### Statistics

For each exposure, gene-environment (GxE) interaction scans were performed using the R software package *GxEScanR* developed by members of our team.^18^ These included traditional 1 degree of freedom analyses to test the multiplicative scale interaction (GxE) using logistic regression models, adjusting for age, sex, and the first three principal components of ancestry, and total energy (only for dietary exposures).

Only SNPs that map onto protein-coding genes within the pathway libraries of interest were brought forward to the pathway analyses. Each SNP was mapped to a protein-encoding gene extended by 20 kb [gene start −20 kb; gene end + 20 kb], using the genomic coordinates obtained from Ensembl's BioMart (assessed: Jan 16, 2022).^19^^,^^20^ This process resulted in approximately 2.8 million SNPs mapping to proteins coding genes. Of these, approximately 2 million SNPs were assigned to 11,441 protein-encoding genes (S.Figure 1), of 2950 canonical pathways described in the gene-set (pathway) libraries of BIOCARTA (n = 292 gene-sets), Kyoto Encyclopedia of Genes and Genomes (KEGG) (n = 186), Pathway Interaction Database (PID) (n = 196), REACTOME (n = 1614) and WikiPathways (n = 662) from the Molecular Signatures Database (MSigDB).^21^, ^22^, ^23^ Details on the different gene-set libraries used can be found in the MSigDB resource and are briefly described in the Methods Supplement.^21^, ^22^, ^23^

We employed two complementary approaches in parallel, each with distinct methodological characteristics, for identifying associated and over-represented pathways linked to the development of CRC: the adaptive combination of Bayes Factors (ADABF) method, and over-representation analysis (ORA). The ADABF tests the association null hypothesis: “*no pathway gene-environment interactions are associated with CRC*”, which is weaker than the competitive null hypothesis typically tested in the ORA: “*the pathway gene-environment interactions are more associated with CRC than genes outside the pathway*”.^24^ The two approaches are described below.

We used the ADABF method proposed by Lin et al., to aggregate the variants’ information within genes and pathways.^24^ In brief, the method uses a four-step procedure to compute adjusted *P*-values, for each gene/pathway. In the first step, approximate Bayes factors (BF) for all the SNPs in the gene/pathway of interest for L variants (SNPs) on a region (gene locus), are estimated: $BF=\sqrt{\frac{\hat{V}}{\hat{V}+W}}\exp\left(\frac{\hat{\beta }W}{2\hat{V}\left(\hat{V}+W\right)}\right)$, where $\hat{\beta }$ and $\hat{V}$, are the beta and its corresponding variance from the interaction models, and W is the prior variance (here we used W = 0.2^2^ = 0.04 as proposed previously^25^). The computed Bayes factors are then ordered as ${BF}_{\left(1\right)}\leq {BF}_{\left(2\right)}\leq \ldots \leq {BF}_{\left(L\right)}$, where the k-th smallest Bayes factor is denoted as ${BF}_{\left(k\right)}$. Based on the ordered Bayes factors, the summary scores (S) are estimated as: $S_{k}=\sum_{i=1}^{L}I\left({BF}_{\left(i\right)}\geq {BF}_{\left(k\right)}\right)\log\left({BF}_{\left(i\right)}\right),k=1,\ldots ,L$, where I(·) is the indicator function. In our work we have followed the sequential resampling procedure described by Liu et al. for assessing the significance of association between each pathway and disease, with the number of sets drawn set to B = 1000. At the end of the procedure, *P*-values of association are computed and reported in the manuscript.^24^

We also performed an ORA to investigate whether genes from pre-defined pathways were presented more than expected in the list of genes per exposure. For each pathway, ORA generates the *P*-value of a hypergeometric test, where the alternative hypothesis indicates that the pathway contains more genes from the gene list (which is formed with the genes that have at least one SNP with *P*-value <5 × 10^−3^) than expected, using the following equation: $p=\sum_{j=n}^{K}\frac{\left(\begin{array}{c} L-M\\ K-j \end{array}\right)\left(\begin{array}{c} M\\ j \end{array}\right)}{\left(\begin{array}{c} L\\ K \end{array}\right)}$, where *K* is the number of genes in the gene list; *L* is the number of genes in the reference set of genes (background), *M* is the number of genes that are annotated to a particular gene-set (pathway), and *n* is the number of genes in the gene list mapping to M. The number of SNPs and genes, per exposure, with *P*-value <5 × 10^−3^ that were used in the ORA, are presented in S.Table 4.

We used R package *clusterProfiler* to perform the ORA analyses.^26^ The settings/parameters that we used in ORA are shown in S.Table 5, and analysis-specific values of K and L are presented in pertinent table summarizing the results of the analysis.

Multiple testing corrections were applied on the generated *P*-values, for each exposure, separately, using the False Discovery Rate (FDR) approach, at the pathway level.^27^ These were largely used to prioritize pathways in the ORA analysis, rather than the ADABF. By aggregating evidence adaptively across genes or variants, the Bayesian framework implemented in the ADABF approach inherently handles multiple testing.

To facilitate the presentation of the results, exposures were grouped by exposure category (S.Table 2) and the minimum *P*-value (across category sub-measurements) per gene/pathway was retained.

Further evidence of association with CRC risk for the genes included in the identified pathways was obtained from different sources. First, we used the gene ontology resource, and previously reported grouping scheme to assign enriched genes to hallmarks of cancer (Activating Invasion and Metastasis, Avoiding Immune Destruction, Deregulating Cellular Metabolism, Enabling Replicative Immortality, Evading Growth Suppressors, Genome Instability and Mutation, Resisting Cell Death, Sustaining Proliferative Signalling, Tumor-promoting Inflammation, Inducing or Accessing Vasculature).^28^, ^29^, ^30^ Second, genes included within previously identified CRC risk loci were obtained from the latest GWASs.^31^ Finally, Overall Association Scores were obtained from the Open Targets Platform (OTP). OTP is a publicly available resource that integrates a wide set of datasets, including genetic associations, somatic mutations, drugs, pathways and systems biology, RNA expression, text mining, and animal models.^32^ Using this information, OTP can score genes with regard to an outcome on a scale of 0–1 (Overall Association Score), with values closer to one indicating stronger evidence. We used “Colorectal neoplasm” as the outcome term and a cut-off of 0.05 (corresponding to approximately the top 20%) to highlight genes with higher *a-priori* evidence.

All analyses were performed using R.^26^^,^^33^

### Ethics

All participants gave written informed consent and studies were approved by their respective Institutional Review Boards.

### Role of funders

The funders had no role in the design of the study; the collection, analysis, and interpretation of the data; the writing of the manuscript; or the decision to submit the manuscript for publication.

## Results

### Overview of associated and enriched pathways and genes

#### Associated pathways (ADABF)

Using the ADABF framework, a total of 1973 pathways were enriched (*P* < 0.05) out of the 2950 that were analysed for at least one exposure (S.Tables 6 and 7). Most enriched pathways were enriched for a single exposure: 136/181 (75.1%) of BIOCARTA, 96/139 (69.1%) of PID, 279/437 (63.8%) of WikiPathways, 667/1082 (61.6%) of REACTOME, and 67/134 (50%) of KEGG pathways. Several pathways were enriched for a combination of two exposures (21.0% of BIOCARTA to 39.6% of KEGG pathways), and few pathways were enriched for a combination of three (3.6% of PID to 10.4 of KEGG pathways) and four exposures (one WikiPathways and four REACTOME pathways). The majority were for combinations of BMI, smoking and NSAIDs (S.Table 8).

#### Enriched pathways (ORA)

Using ORA, 840 unique pathways, out of 2,950, were enriched (*P* < 0.05) across exposures and genes-sets, less than half of which for a single exposure: 20/37 (54.1%) of BIOCARTA, 37/88 (42.0%) of PID, 84/227 (37.0%) of WikiPathways, 154/385 (40.0%) of REACTOME, and 23/103 (22.3%) of KEGG pathways (S.Table 9).

#### Associated genes (ADABF)

**Fig. 2:**
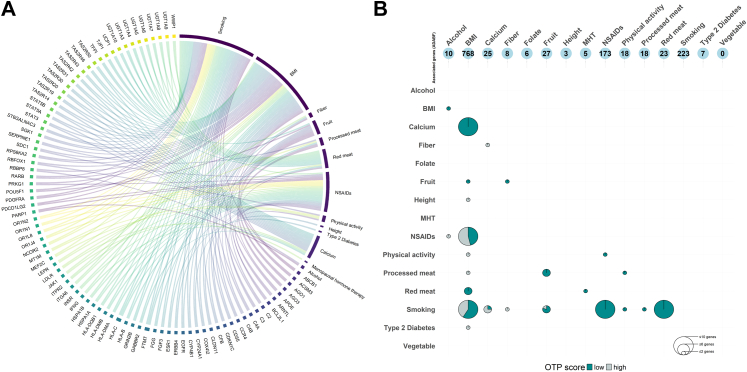
Summary of enriched genes (*P* < 0.05) and genes commonly enriched across exposures using the adaptive combination of Bayes factors approach. **A.** Commonly enriched genes across different exposures. **B.** Total genes enriched per exposure (top), and number overlapping genes across exposures, by Open targets platform (OTP) score. BMI: Body mass index; NSAIDs: non-steroidal anti-inflammatory drugs. **List of gene abbreviations on the plot:** ABCB1: ATP binding cassette subfamily B member 1; ACSM3: acyl-CoA synthetase medium chain family member 3; AGO1: argonaute RISC component 1; AGO3: argonaute RISC catalytic component 3; APOE: apolipoprotein E; ARNTL: basic helix-loop-helix ARNT like 1; BCL2L1: BCL2 like 1; C2: complement C2; C3: complement C3; C4A: complement C4A (Chido/Rodgers blood group); C4B: complement C4B (Chido/Rodgers blood group); CCR4: C–C motif chemokine receptor 4; CD55: CD55 molecule (Cromer blood group); CDKN1C: cyclin dependent kinase inhibitor 1C; CFB: complement factor B; CLDN11: claudin 11; COX4I2: cytochrome *c* oxidase subunit 4I2; CYP24A1: cytochrome P450 family 24 subfamily A member 1; CYP4B1: cytochrome P450 family 4 subfamily B member 1; EGFR: epidermal growth factor receptor; ERBB4: erb-b2 receptor tyrosine kinase 4; ESR1: estrogen receptor 1; FGF3: fibroblast growth factor 3; FGG: fibrinogen gamma chain; FTMT: ferritin mitochondrial; GABBR2: gamma-aminobutyric acid type B receptor subunit 2; GRIN2B: glutamate ionotropic receptor NMDA type subunit 2B; HLA-B: major histocompatibility complex, class I, B; HLA-C: major histocompatibility complex, class I, C; HLA-DMA: major histocompatibility complex, class II, DM alpha; HLA-DMB: major histocompatibility complex, class II, DM beta; HLA-DQB1: major histocompatibility complex, class II, DQ beta 1; HSPA1A: heat shock protein family A (Hsp70) member 1A; HSPA1B: heat shock protein family A (Hsp70) member 1B; IFNG: interferon gamma; INSR: insulin receptor; ITGA6: integrin subunit alpha 6; ITPR2: inositol 1,4,5-trisphosphate receptor type 2; JAK1: Janus kinase 1; LDLR: low density lipoprotein receptor; LEPR: leptin receptor; MEF2C: myocyte enhancer factor 2C; MT1M: metallothionein 1M; NCOR2: nuclear receptor corepressor 2; OR1J4: olfactory receptor family 1 subfamily J member 4; OR1L8: olfactory receptor family 1 subfamily L member 8; OR1N1: olfactory receptor family 1 subfamily N member 1; OR1N2: olfactory receptor family 1 subfamily N member 2; PARP1: poly(ADP-ribose) polymerase 1; PDCD1LG2: programmed cell death 1 ligand 2; PDGFRA: platelet derived growth factor receptor alpha; POU5F1: POU class 5 homeobox 1; PRKG1: protein kinase cGMP-dependent 1; RARB: retinoic acid receptor beta; RBBP8: RB binding protein 8, endonuclease; RBFOX1: RNA binding fox-1 homolog 1; RPS6KA2: ribosomal protein S6 kinase A2; SDC1: syndecan 1; SERPINE1: serpin family E member 1; SGK1: serum/glucocorticoid regulated kinase 1; ST6GALNAC3: ST6 N-acetylgalactosaminide alpha-2,6-sialyltransferase 3; STAT3: signal transducer and activator of transcription 3; STAT5A: signal transducer and activator of transcription 5A; STAT5B: signal transducer and activator of transcription 5B; TAS2R14: taste 2 receptor member 14; TAS2R19: taste 2 receptor member 19; TAS2R20: taste 2 receptor member 20; TAS2R30: taste 2 receptor member 30; TAS2R31: taste 2 receptor member 31; TAS2R42: taste 2 receptor member 42; TAS2R43: taste 2 receptor member 43; TAS2R46: taste 2 receptor member 46; TAS2R50: taste 2 receptor member 50; TFPI: tissue factor pathway inhibitor; TJP1: tight junction protein 1; UCP1: uncoupling protein 1; UGT1A10: UDP glucuronosyltransferase family 1 member A10; UGT1A3: UDP glucuronosyltransferase family 1 member A3; UGT1A4: UDP glucuronosyltransferase family 1 member A4; UGT1A5: UDP glucuronosyltransferase family 1 member A5; UGT1A6: UDP glucuronosyltransferase family 1 member A6; UGT1A7: UDP glucuronosyltransferase family 1 member A7; UGT1A8: UDP glucuronosyltransferase family 1 member A8; UGT1A9: UDP glucuronosyltransferase family 1 member A9; WWP1: WW domain containing E3 ubiquitin protein ligase 1.

Within the ADABF enriched pathways, a total of 1227 genes were associated (*P* < 0.05) with at least one exposure. Most of which were found for BMI (n = 768), followed by smoking (n = 223), NSAIDs (n = 173), fruit (n = 27), calcium (n = 25), red meat (n = 23), processed meat (n = 18), physical activity (n = 18), alcohol (n = 10), fibre (n = 8), T2D (n = 7), folate (n = 6), MHT (n = 5), and height (n = 3) (S.Table 10). Most of the genes (1142/1227; 93%) were associated with a single exposure, and 85 were associated with multiple exposures, the majority of which for combinations of BMI, smoking and NSAIDs (Fig. 2 and S.Table 11).

A total of 59 genes with a relatively lower a priori evidence for association with CRC (with an OTP ≤0.05 or not in the OTP database), were found to be enriched for any combination of exposures (Fig. 2 and S.Tables 10 and 11). Extensive overlaps, covering a wide range of genes [including cytochrome P450 family 4 subfamily B member 1 (*CYP4B1*), fibroblast growth factor 3 (*FGF3*), *GRIN2B*, integrin subunit alpha 6 (*ITGA6*), uncoupling protein 1 (*UCP1*), etc.] were found for BMI, and smoking. A similarly wide range of genes was enriched for BMI, and NSAIDs [such as inositol 1,4,5-trisphosphate receptor type 2 (*ITPR2*), protein kinase cGMP-dependent 1 (*PRKG1*), complement C3 (*C3*), claudin 11 (*CLDN11*), etc.]. Immune and stress-response related genes (such as Major Histocompatibility Complex coding genes, and members of the Heat Shock Protein family), as well as several genes of the Olfactory Receptor family [such as olfactory receptor family 1 subfamily N member 1 (*OR1N1*)] were commonly enriched for NSAIDs, and smoking. Multiple genes of the UDP glucuronosyltransferase family [such as UDP glucuronosyltransferase family 1 member A10 (*UGT1A10*)] involved in detoxification pathways were enriched for red meat intake and smoking. Among the overlaps were also genes associated with lipid metabolism, such as apolipoprotein E (*APOE*; enriched for red meat intake and smoking), acyl-CoA synthetase medium chain family member 3 (*ACSM3*; enriched for intakes of fibre and fruit), and low-density lipoprotein receptor (*LDLR*; enriched for MHT and red meat intake). Argonaute RISC component genes (such as *AGO1*, *AGO3*) involved in gene silencing were enriched for intakes of fruit and processed meat. Several taste receptor genes [such as taste 2 receptor member 20 (*TAS2R20*)], as well as gamma-aminobutyric acid type B receptor subunit 2 (*GABBR2*) were commonly enriched for BMI, and calcium intake. Several complement-related genes were enriched for fruit intake and smoking. Enrichment of *ARNTL* (basic helix-loop-helix ARNT like 1), implicated in circadian rhythms, was observed for NSAIDs and physical activity. Other enriched genes include: ferritin mitochondrial (*FTMT*; BMI, smoking, red meat), fibrinogen gamma chain (*FGG*; enriched for smoking, and processed meat intake), metallothionein 1M (*MT1M*; processed meat, physical activity), tight junction protein 1 (*TJP1*; BMI, fruit), tissue factor pathway inhibitor (*TFPI*; BMI, red meat), RB binding protein 8, endonuclease (*RBBP8*; BMI, alcohol), myocyte enhancer factor 2C (*MEF2C*; smoking, physical activity), and signal transducer and activator of transcription 5A (*STAT5A*; smoking and calcium).

The number of enriched genes within enriched pathways (ADABF) ranged from a minimum of one, to a maximum of 111, depending on the database, with a maximum of eight for BIOCARTA, 13 for PID, 37 for KEGG, 38 for WikiPathways, 111 for REACTOME (S.Table 12).

### Highlighted pathways and genes

#### Top pathways

**Fig. 3:**
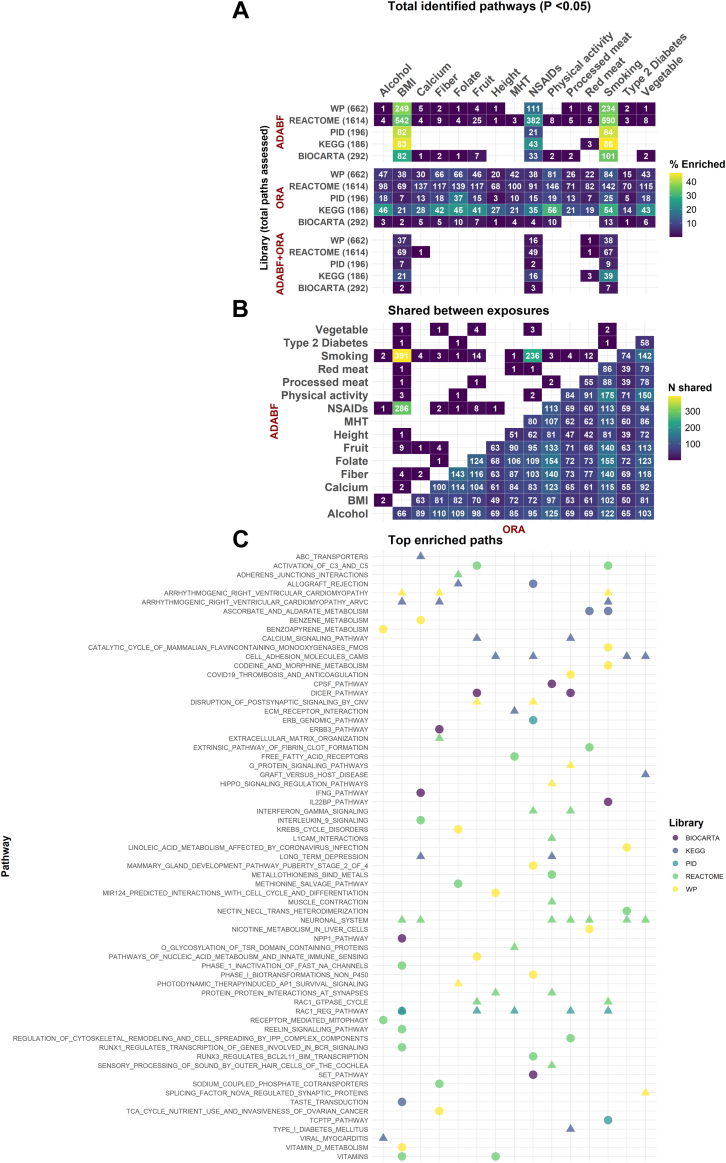
Summary of enriched (*P* < 0.05) pathways. **A.** Total number of enriched pathways per gene-set library, exposure, and method used. Color is analogous to the percent of pathways enriched relative to pathways assessed. **B.** Overlapping of enriched pathways across exposures, per method used. **C.** Top enriched pathway per gene-set library, and exposure. Circles represent associated ADABF pathways with the size analogous to the percent of enriched genes within the pathway, and triangles represent enriched ORA pathways. The top ADABF pathways were defined by the total number of enriched genes within the pathway, while top ORA pathways based on the minimum q-value. ADABF: adaptive combination of Bayes factors; KEGG: Kyoto Encyclopedia of Genes and Genomes; ORA: Over-representation analysis; PID: Pathway Interaction Database; SNPs: Single nucleotide polymorphisms; WP: WikiPathways.

The top pathways based on the total number of enriched genes within the pathway, using the ADABF approach, are shown in Fig. 3. Seven hundred and fifty-two unique pathways had an FDR<5%, all across BMI (n = 688), NSAIDs (n = 68), and smoking (n = 53), the majority of which (698/752; 92.8%) for a single exposure (S.Table 7).

Using ORA, 152 unique pathways had an FDR<5% (S.Table 9). These pathways belonged to the libraries of KEGG (54/152; 35.5%), REACTOME (80/152; 52.6%), WikiPathways (16/152; 10.5%), and PID (2/152; 1.3%). Across gene-set libraries, 65 out of the 152 enriched pathways (42.8%) were below the 5% FDR threshold for a single exposure: 16/54 (29.6%) of KEGG, 40/80 (50.0%) of REACTOME, and 9/16 (56.3%) of WikiPathways. Thirty two of the 152 pathways (21.1%) had FDR < 5% for any combination of two exposures, and one pathway (REACTOME's *Neuronal system*) passed the 5% threshold for 14 different exposures (BMI, smoking, calcium, T2D, fibre, folate, fruit, height, MHT, NSAIDs, PA, processed meat, red meat, vegetables) (S.Table 9). Top pathways based on the minimum q-value are shown in Fig. 3.

A total of 297 pathways across five exposures (BMI, NSAIDs, smoking, calcium, and red meat) were enriched (*P* < 0.05) using both the ADABF and ORA approaches (S.Table 13). These pathways pertained to intra- and inter-cellular communication (such as the ErbB, Notch, MAPK, and epidermal growth factor (EGF) signalling pathways, and G-protein-mediated cascades), extracellular matrix organization, and cell adhesion [represented through pathways such as extracellular matrix organization, collagen biosynthesis and degradation, focal adhesion, and cell adhesion molecules (CAMs)], as well as regulation of the cytoskeleton (such as Integrin cell surface interactions). Immune Response and Inflammation (including interferon signalling, antigen processing and presentation), hormonal regulation pathways (such as aldosterone-regulated sodium reabsorption, insulin secretion and processing, and progesterone-mediated oocyte maturation), and pathways related to metabolism and biosynthetic processes (such as steroid hormone biosynthesis, glycosaminoglycan biosynthesis, and metabolism of steroids and fat-soluble vitamins), were also among the pathways that were identified using both ADABF and ORA approaches. Additionally, pathways involved in the nervous system (including those regulating neurotransmitter receptors and synaptic signalling, long-term potentiation and depression, and glutamate and GABA receptor activation), and pathways associated with cardiovascular and muscular functions, such as calcium signalling pathways, as well as hypertrophic and dilated cardiomyopathy pathways and cardiac conduction and contraction pathways were also included. Developmental pathways were also included, pertaining to processes such as nervous system development, neural crest cell migration, and pathways related to ectoderm and mesoderm development. Malignancy-associated pathways, such as pathways in cancer, small-cell and non-small-cell lung cancer, and melanoma, and other disease-specific pathways such as systemic lupus erythematosus and graft-versus-host disease, type I and type II diabetes mellitus, Alzheimer's disease, Rett syndrome were also in the list of commonly enriched pathways using the two approaches.

#### Top associated genes

Data were available for 811/1227 genes in the OTP; an overall association score >0.05 was found for 241 coding genes (S.Table 10). Most of the genes with score >0.05 were found for BMI (153/241), followed by smoking (47/241), NSAIDs (41/241), T2D (6/241), calcium (5/241, each), fruit (3/241), PA, alcohol, height, fibre, processed meat (2/241, each), and MHT (1/241).

Fifty percent of the genes (617/1227) mapped to at least one hallmark of cancer across schemes, most of which (388/617) pertained to the *Sustaining Proliferative Signalling* hallmark (S.Table 14). Exposure-wise, *Activating Invasion and Metastasis* was the top contributing hallmark for BMI (69% of genes), Avoiding Immune Destruction was the top contributing hallmark for smoking (28%) and NSAIDs (39%), and *Evading Growth Suppressors* was the top contributing hallmark for Calcium (5%), excluding categories with <5 participating genes.

Nine of the 1227 genes were previously identified as genome-wide significant CRC risk loci: DLG associated protein 1 (*DLGAP1*), pericentrin (*PCNT*), signal peptide, CUB domain and EGF like domain containing 1 (*SCUBE1*), vav guanine nucleotide exchange factor 2 (*VAV2*), Ras And Rab Interactor 3 (*RIN3*), Collagen Type IV Alpha 2 Chain (*COL4A2*), and Vesicle Transport Through Interaction With T-SNAREs 1A (*VTI1A*) enriched for BMI, Major Histocompatibility Complex, Class I, B (*HLA-B*) enriched for NSAIDs, Notch Receptor 4 (*NOTCH4*) enriched for fruit^15^^,^^31^^,^^34^, ^35^, ^36^(S.Table 10).

Nearly half of the associated genes (689/1227; 56.2%) participated in pathways that were found to be enriched using both ADABF and ORA approaches, 177 of which (177/689; 25.7%) had an overall OTP score >0.05 (S.Table 13).

**Fig. 4:**
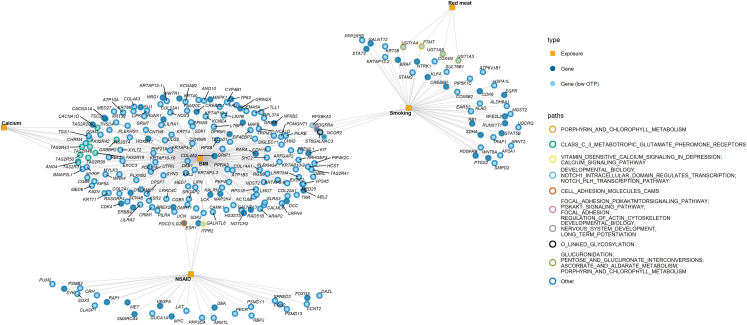
Network of genes identified using the adaptive combination of Bayes factors (ADABF) within pathways that were highlighted by both the ADABF and over-representation analysis (ORA). The plot displays pathways containing genes commonly enriched across any combination of exposures. Human Leukocyte Antigen (HLA) genes were not included in the network. BMI: Body mass index; NSAIDs: non-steroidal anti-inflammatory drugs; OTP: open targets platform. **List of gene abbreviations on the plot:** ABL2: ABL proto-oncogene 2, non-receptor tyrosine kinase; ACTL6B: actin like 6B; ALAD: aminolevulinate dehydratase; ALDH9A1: aldehyde dehydrogenase 9 family member A1; ANO10: anoctamin 10; ANO4: anoctamin 4; AP2A1: adaptor related protein complex 2 subunit alpha 1; ARAP2: ArfGAP with RhoGAP domain, ankyrin repeat and PH domain 2; ARFGAP2: ADP ribosylation factor GTPase activating protein 2; ARHGEF3: Rho guanine nucleotide exchange factor 3; ARL2: ADP ribosylation factor like GTPase 2; ARNT2: aryl hydrocarbon receptor nuclear translocator 2; ATP10A: ATPase phospholipid transporting 10A (putative); ATP1B3: ATPase Na+/K+ transporting subunit beta 3; ATP6V1B1: ATPase H+ transporting V1 subunit B1; BAIAP2L1: BAR/IMD domain containing adaptor protein 2 like 1; ARNTL: basic helix-loop-helix ARNT like 1; BRAF: B-Raf proto-oncogene, serine/threonine kinase; CACNA1A: calcium voltage-gated channel subunit alpha1 A; CACNA1D: calcium voltage-gated channel subunit alpha1 D; CALML6: calmodulin like 6; CAMK1: calcium/calmodulin dependent protein kinase I; CCNT2: cyclin T2; CDH18: cadherin 18; CDK4: cyclin dependent kinase 4; CDK6: cyclin dependent kinase 6; CGB5: chorionic gonadotropin subunit beta 5; CGB8: chorionic gonadotropin subunit beta 8; CHN2: chimerin 2; CHRM4: cholinergic receptor muscarinic 4; CLASP1: cytoplasmic linker associated protein 1; CNTN6: contactin 6; COL14A1: collagen type XIV alpha 1 chain; COL23A1: collagen type XXIII alpha 1 chain; COL25A1: collagen type XXV alpha 1 chain; COL2A1: collagen type II alpha 1 chain; COL4A2: collagen type IV alpha 2 chain; COL4A3: collagen type IV alpha 3 chain; COX4I2: cytochrome *c* oxidase subunit 4I2; COX6B2: cytochrome *c* oxidase subunit 6B2; CPSF7: cleavage and polyadenylation specific factor 7; CREB3L1: cAMP responsive element binding protein 3 like 1; CREBBP: CREB binding protein; CRH: corticotropin releasing hormone; CRIM1: cysteine rich transmembrane BMP regulator 1; CYP4B1: cytochrome P450 family 4 subfamily B member 1; DAB1: DAB adaptor protein 1; DAZL: deleted in azoospermia like; DBNL: drebrin like; DCC: DCC netrin 1 receptor; DEK: DEK proto-oncogene; DLG2: discs large MAGUK scaffold protein 2; DMRT1: doublesex and mab-3 related transcription factor 1; DNM3: dynamin 3; DSCAML1: DS cell adhesion molecule like 1; EARS2: glutamyl-tRNA synthetase 2, mitochondrial; EFNA5: ephrin A5; EGFR: epidermal growth factor receptor; EIF4EBP2: eukaryotic translation initiation factor 4E binding protein 2; ERBB4: erb-b2 receptor tyrosine kinase 4; ESR1: estrogen receptor 1; EXOC2: exocyst complex component 2; EXOC3: exocyst complex component 3; FAM20C: FAM20C golgi associated secretory pathway kinase; FAU: FAU ubiquitin like and ribosomal protein S30 fusion; FLI1: Fli-1 proto-oncogene, ETS transcription factor; FOXO3: forkhead box O3; FTMT: ferritin mitochondrial; GABBR2: gamma-aminobutyric acid type B receptor subunit 2; GALNT12: polypeptide N-acetylgalactosaminyltransferase 12; GALNT13: polypeptide N-acetylgalactosaminyltransferase 13; GALNTL6: polypeptide N-acetylgalactosaminyltransferase like 6; GLRA3: glycine receptor alpha 3; GRIK2: glutamate ionotropic receptor kainate type subunit 2; GRIN2A: glutamate ionotropic receptor NMDA type subunit 2A; GRIP1: glutamate receptor interacting protein 1; GRM7: glutamate metabotropic receptor 7; GUCA1A: guanylate cyclase activator 1A; HCST: hematopoietic cell signal transducer; HOXD11: homeobox D11; HS3ST4: heparan sulfate-glucosamine 3-sulfotransferase 4; HS3ST5: heparan sulfate-glucosamine 3-sulfotransferase 5; HSPA1L: heat shock protein family A (Hsp70) member 1 like; ITPR2: inositol 1,4,5-trisphosphate receptor type 2; KALRN: kalirin RhoGEF kinase; KAZN: kazrin, periplakin interacting protein; KCNAB2: potassium voltage-gated channel subfamily A regulatory beta subunit 2; KCNE4: potassium voltage-gated channel subfamily E regulatory subunit 4; KIF5A: kinesin family member 5A; KIT: KIT proto-oncogene, receptor tyrosine kinase; KLF4: KLF transcription factor 4; KRT38: keratin 38; KRT39: keratin 39; KRT40: keratin 40; KRT71: keratin 71; KRT74: keratin 74; KRT86: keratin 86; KRTAP10-10: keratin associated protein 10-10; KRTAP10-4: keratin associated protein 10-4; KRTAP10-9: keratin associated protein 10-9; KRTAP12-1: keratin associated protein 12-1; KRTAP12-2: keratin associated protein 12-2; KRTAP3-2: keratin associated protein 3-2; KRTAP3-3: keratin associated protein 3-3; KRTAP5-6: keratin associated protein 5-6; KSR2: kinase suppressor of ras 2; LAT: linker for activation of T cells; LCK: LCK proto-oncogene, Src family tyrosine kinase; LILRA1: leukocyte immunoglobulin like receptor A1; LILRA2: leukocyte immunoglobulin like receptor A2; LIN7B: lin-7 homolog B, crumbs cell polarity complex component; LIPK: lipase family member K; LIPN: lipase family member N; LMO7: LIM domain 7; LRFN4: leucine rich repeat and fibronectin type III domain containing 4; LRRC4C: leucine rich repeat containing 4C; LRRTM4: leucine rich repeat transmembrane neuronal 4; LTBP1: latent transforming growth factor beta binding protein 1; MAFB: MAF bZIP transcription factor B; MAP2K4: mitogen-activated protein kinase kinase 4; MAPK1: mitogen-activated protein kinase 1; MED25: mediator complex subunit 25; MED27: mediator complex subunit 27; MED6: mediator complex subunit 6; MED7: mediator complex subunit 7; MET: MET proto-oncogene, receptor tyrosine kinase; MGST2: microsomal glutathione S-transferase 2; MYC: MYC proto-oncogene, bHLH transcription factor; MYLK3: myosin light chain kinase 3; MYRIP: myosin VIIA and Rab interacting protein; NCALD: neurocalcin delta; NCOR2: nuclear receptor corepressor 2; NDST2: N-deacetylase and N-sulfotransferase 2; NFE2L2: NFE2 like bZIP transcription factor 2; NFKB2: nuclear factor kappa B subunit 2; NOTCH2: notch receptor 2; NOX3: NADPH oxidase 3; NRG1: neuregulin 1; NTRK1: neurotrophic receptor tyrosine kinase 1; OPRM1: opioid receptor mu 1; PDCD1LG2: programmed cell death 1 ligand 2; PDE11A: phosphodiesterase 11A; PDGFRA: platelet derived growth factor receptor alpha; PDGFRB: platelet derived growth factor receptor beta; PECR: peroxisomal trans-2-enoyl-CoA reductase; PHF21A: PHD finger protein 21A; PILRA: paired immunoglobin like type 2 receptor alpha; PILRB: paired immunoglobin like type 2 receptor beta; PIP4K2C: phosphatidylinositol-5-phosphate 4-kinase type 2 gamma; PIP5K1C: phosphatidylinositol-4-phosphate 5-kinase type 1 gamma; PKP4: plakophilin 4; PLEKHG1: pleckstrin homology and RhoGEF domain containing G1; PLEKHG4: pleckstrin homology and RhoGEF domain containing G4; PLXNB2: plexin B2; POMGNT1: protein O-linked mannose N-acetylglucosaminyltransferase 1 (beta 1,2-); PON2: paraoxonase 2; PPFIA3: PTPRF interacting protein alpha 3; PPP2R5B: protein phosphatase 2 regulatory subunit B'beta; PPP3CA: protein phosphatase 3 catalytic subunit alpha; PREX2: phosphatidylinositol-3,4,5-trisphosphate dependent Rac exchange factor 2; PSMB3: proteasome 20S subunit beta 3; PSMD11: proteasome 26S subunit, non-ATPase 11; PSMD13: proteasome 26S subunit, non-ATPase 13; PTGS2: prostaglandin-endoperoxide synthase 2; PUM2: pumilio RNA binding family member 2; RAD51B: RAD51 paralog B; RAF1: Raf-1 proto-oncogene, serine/threonine kinase; RARA: retinoic acid receptor alpha; RASGRP4: RAS guanyl releasing protein 4; RB1: RB transcriptional corepressor 1; RBL2: RB transcriptional corepressor like 2; RBPJ: recombination signal binding protein for immunoglobulin kappa J region; RGS6: regulator of G protein signalling 6; RPL37A: ribosomal protein L37a; RPS18: ribosomal protein S18; RPS6KA2: ribosomal protein S6 kinase A2; RPS9: ribosomal protein S9; RUNX1: RUNX family transcription factor 1; RUNX1T1: RUNX1 partner transcriptional co-repressor 1; SCN4B: sodium voltage-gated channel beta subunit 4; SDHA: succinate dehydrogenase complex flavoprotein subunit A; SDK1: sidekick cell adhesion molecule 1; SDK2: sidekick cell adhesion molecule 2; SEMA5A: semaphorin 5A; SHC2: SHC adaptor protein 2; SIGLEC11: sialic acid binding Ig like lectin 11; SMARCA4: SWI/SNF related, matrix associated, actin dependent regulator of chromatin, subfamily a, member 4; SMPD2: sphingomyelin phosphodiesterase 2; SOX5: SRY-box transcription factor 5; SPRED2: sprouty related EVH1 domain containing 2; SRGAP2: SLIT-ROBO Rho GTPase activating protein 2; ST6GALNAC3: ST6 N-acetylgalactosaminide alpha-2,6-sialyltransferase 3; STAM2: signal transducing adaptor molecule 2; STAT3: signal transducer and activator of transcription 3; STAT5B: signal transducer and activator of transcription 5B; SULT6B1: sulfotransferase family 6B member 1; SYK: spleen associated tyrosine kinase; SYN3: synapsin III; TAS2R19: taste 2 receptor member 19; TAS2R20: taste 2 receptor member 20; TAS2R30: taste 2 receptor member 30; TAS2R31: taste 2 receptor member 31; TAS2R41: taste 2 receptor member 41; TAS2R42: taste 2 receptor member 42; TAS2R43: taste 2 receptor member 43; TAS2R46: taste 2 receptor member 46; TAS2R50: taste 2 receptor member 50; TBC1D1: TBC1 domain family member 1; TGS1: trimethylguanosine synthase 1; THSD4: thrombospondin type 1 domain containing 4; TLL1: tolloid like 1; TNR: tenascin R; TP63: tumour protein p63; TRAF1: TNF receptor associated factor 1; TREML1: triggering receptor expressed on myeloid cells like 1; TRPM3: transient receptor potential cation channel subfamily M member 3; TSC2: TSC complex subunit 2; UCN: urocortin; UGT1A3: UDP glucuronosyltransferase family 1 member A3; UGT1A4: UDP glucuronosyltransferase family 1 member A4; UGT1A5: UDP glucuronosyltransferase family 1 member A5; UQCRQ: ubiquinol-cytochrome c reductase complex III subunit VII; UTRN: utrophin; VEGFA: vascular endothelial growth factor A; VPS45: vacuolar protein sorting 45 homolog; WNK4: WNK lysine deficient protein kinase 4; WNT8A: Wnt family member 8A; WWTR1: WW domain containing transcription regulator 1; XYLT2: xylosyltransferase 2; ZSWIM8: zinc finger SWIM-type containing 8.

**Fig. 5:**
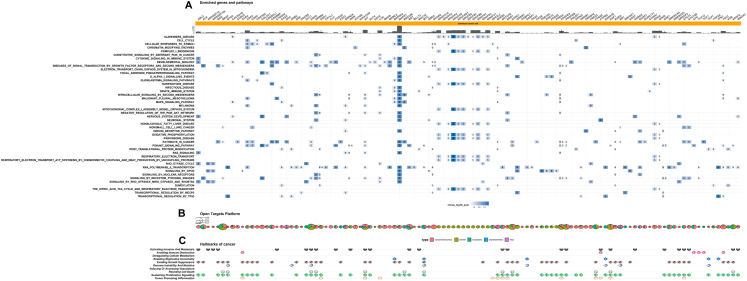
Summary of the enriched genes and pathways using the adaptive combination of Bayes factors (ADABF) approach. Only the genes with an Open Targets Platform (OTP) overall association score >0.2, and pertinent pathways with >5 enriched genes with OTP>0.2 are plotted. **A**. Enriched genes and pathways. X-axis (top) presents enriched genes. Y-axis of the main panel shows the enriched pathways, and the tiles represent the exposures [B: Body mass Index (BMI); S: Smoking: N: Non-Steroidal Anti-inflammatory Drugs (NSAIDs)]. Tile color is analogous to the -log10 ADABF *P*-value of pertinent gene. **B**. Summary of the evidence from the OTP. Each pie-chart represents a gene and illustrates the relative contribution of individual components that form the overall OTP association score, and their size is analogous to the OTP overall association score. Five sub-components of OTP are plotted, cancerGeneCensus: representing genes containing mutations that have been causally implicated in cancer, chembl: representing bioactive molecules with drug-like properties, europepmc: representing gene-colorectal cancer (CRC) co-occurrences in the literature, expressionAtlas: representing differentially expressed genes in CRC, impc: representing the similarity between a mouse model and a human CRC on each gene. **C**. Summary of the Hallmarks of cancer. The bottom panel shows the hallmark capabilities in which each gene has been implicated in based on various grouping schemes. **List of gene abbreviations on the plot:** ABL2: ABL proto-oncogene 2, non-receptor tyrosine kinase; ALK: ALK receptor tyrosine kinase; ARHGEF10: Rho guanine nucleotide exchange factor 10; ARHGEF10L: Rho guanine nucleotide exchange factor 10 like; BCR: BCR activator of RhoGEF and GTPase; BRAF: B-Raf proto-oncogene, serine/threonine kinase; BTG2: BTG anti-proliferation factor 2; CACNA1D: calcium voltage-gated channel subunit alpha1 D; CCDC6: coiled-coil domain containing 6; CCR4: C–C motif chemokine receptor 4; CDK4: cyclin dependent kinase 4; CDK6: cyclin dependent kinase 6; CDKN2C: cyclin dependent kinase inhibitor 2C; COL2A1: collagen type II alpha 1 chain; CREB3L1: cAMP responsive element binding protein 3 like 1; CREBBP: CREB binding protein; CTNNA2: catenin alpha 2; DAXX: death domain associated protein; DCC: DCC netrin 1 receptor; DDB2: damage specific DNA binding protein 2; DEK: DEK proto-oncogene; DGCR8: DGCR8 microprocessor complex subunit; DLC1: DLC1 Rho GTPase activating protein; EGFR: epidermal growth factor receptor; ERBB4: erb-b2 receptor tyrosine kinase 4; ESR1: estrogen receptor 1; FIP1L1: factor interacting with PAPOLA and CPSF1; FLI1: Fli-1 proto-oncogene, ETS transcription factor; FOXO3: forkhead box O3; FZD3: frizzled class receptor 3; GRIN2A: glutamate ionotropic receptor NMDA type subunit 2A; INSR: insulin receptor; KAT6B: lysine acetyltransferase 6B; KIAA1549: KIAA1549; KIT: KIT proto-oncogene, receptor tyrosine kinase; KLF4: KLF transcription factor 4; KRT8: keratin 8; LCK: LCK proto-oncogene, Src family tyrosine kinase; MAFB: MAF bZIP transcription factor B; MAML2: mastermind like transcriptional coactivator 2; MAP2K4: mitogen-activated protein kinase kinase 4; MAPK1: mitogen-activated protein kinase 1; MECOM: MDS1 and EVI1 complex locus; MET: MET proto-oncogene, receptor tyrosine kinase; MLLT3: MLLT3 super elongation complex subunit; MUC16: mucin 16, cell surface associated; MYC: MYC proto-oncogene, bHLH transcription factor; NBEA: neurobeachin; NCOR2: nuclear receptor corepressor 2; NDUFA10: NADH:ubiquinone oxidoreductase subunit A10; NDUFA3: NADH:ubiquinone oxidoreductase subunit A3; NDUFA4: NDUFA4 mitochondrial complex associated; NDUFA8: NADH:ubiquinone oxidoreductase subunit A8; NDUFAB1: NADH:ubiquinone oxidoreductase subunit AB1; NDUFB2: NADH:ubiquinone oxidoreductase subunit B2; NDUFB7: NADH:ubiquinone oxidoreductase subunit B7; NDUFB9: NADH:ubiquinone oxidoreductase subunit B9; NDUFS1: NADH:ubiquinone oxidoreductase core subunit S1; NDUFS3: NADH:ubiquinone oxidoreductase core subunit S3; NDUFV1: NADH:ubiquinone oxidoreductase core subunit V1; NFE2L2: NFE2 like bZIP transcription factor 2; NFKB2: nuclear factor kappa B subunit 2; NOTCH2: notch receptor 2; NRG1: neuregulin 1; NTRK1: neurotrophic receptor tyrosine kinase 1; OPRM1: opioid receptor mu 1; PABPC1: poly(A) binding protein cytoplasmic 1; PARP1: poly(ADP-ribose) polymerase 1; PAX5: paired box 5; PAX8: paired box 8; PDE10A: phosphodiesterase 10A; PDE1C: phosphodiesterase 1C; PDE4D: phosphodiesterase 4D; PDE8A: phosphodiesterase 8A; PDGFRA: platelet derived growth factor receptor alpha; PDGFRB: platelet derived growth factor receptor beta; POU5F1: POU class 5 homeobox 1; PPP6C: protein phosphatase 6 catalytic subunit; PRDM1: PR/SET domain 1; PRDM16: PR/SET domain 16; PREX2: phosphatidylinositol-3,4,5-trisphosphate dependent Rac exchange factor 2; PTGS2: prostaglandin-endoperoxide synthase 2; PTPRD: protein tyrosine phosphatase receptor type D; RAD51D: RAD51 paralog D; RAF1: Raf-1 proto-oncogene, serine/threonine kinase; RARA: retinoic acid receptor alpha; RB1: RB transcriptional corepressor 1; RBFOX1: RNA binding fox-1 homolog 1; RGS7: regulator of G protein signalling 7; ROBO2: roundabout guidance receptor 2; RUNX1: RUNX family transcription factor 1; RUNX1T1: RUNX1 partner transcriptional co-repressor 1; S100A7: S100 calcium binding protein A7; SDHA: succinate dehydrogenase complex flavoprotein subunit A; SDHD: succinate dehydrogenase complex subunit D; SGK1: serum/glucocorticoid regulated kinase 1; SMARCA4: SWI/SNF related, matrix associated, actin dependent regulator of chromatin, subfamily a, member 4; SMARCB1: SWI/SNF related, matrix associated, actin dependent regulator of chromatin, subfamily b, member 1; SRGAP3: SLIT-ROBO Rho GTPase activating protein 3; STAT3: signal transducer and activator of transcription 3; STAT5B: signal transducer and activator of transcription 5B; SYK: spleen associated tyrosine kinase; TCF12: transcription factor 12; TCF7: transcription factor 7; TFPT: TCF3 fusion partner; TOP2A: DNA topoisomerase II alpha; TP63: tumour protein p63; TSC2: TSC complex subunit 2; VDR: vitamin D receptor; VEGFA: vascular endothelial growth factor A; WWTR1: WW domain containing transcription regulator 1.

A selection of genes—and the pathways in which they are involved—including those not represented in the platform (to highlight potentially understudied associations) as well as genes with high OTP scores, is presented in Figs. 4 and 5.

## Discussion

By utilizing large scale genome-wide gene-environment interaction estimates for 15 different exposures, we agnostically investigated the molecular pathways underlying risk of CRC. Our analysis provides evidence to support the implication of several pathways and participating genes, few of which were previously identified as genome-wide significant CRC risk loci, potentially adding to the existing knowledge underlying the mechanisms behind CRC risk. Additionally, among the implicated genes and pertinent molecular pathways, concomitantly enriched pathways and genes for different exposures are highlighted, potentially suggesting common underlying mechanisms. The results of the present analysis provide a basis for further functional investigation. If confirmed, these findings could contribute to the development of personalized CRC prevention strategies. They could be used to inform refined risk stratification models that incorporate gene–environment interactions. Identifying genes and pathways whose effects are modifiable by lifestyle or environmental factors paves the way for targeted interventions aimed at mitigating genetic risk through behavioural or environmental modification.

Accumulating evidence over the past decades has established the importance of signalling pathways such as MAPK, Notch, PI3K/AKT pathway, TGF-β, and Wnt, in cell proliferation, differentiation, angiogenesis, invasion, apoptosis and survival in CRC.^37^ Our analysis confirmed the above pathways and participating genes, several of which were overrepresented for exposures, such as smoking, BMI and NSAIDs. For instance, differential associations between smoking and CRC risk have been observed by pertinent categories of established molecular subtypes of CRC, such as mutations of B-Raf Proto-Oncogene, Serine/Threonine Kinase (*BRAF*) and KRAS Proto-Oncogene, GTPase (*KRAS*), CpG island methylator phenotype (CIMP), and microsatellite instability (MSI).^38^ Genes closely linked to those molecular subtypes, such as *BRAF*, CREB Binding Protein (*CREBBP*), Epidermal Growth Factor Receptor (*EGFR*), Platelet Derived Growth Factor Receptor Beta (*PDGFRB*) and Neurotrophic Receptor Tyrosine Kinase 1 (*NTRK1*), were highlighted in interaction with smoking.^38^, ^39^, ^40^, ^41^ Additionally, genes that are known for their crucial role in carcinogenesis, such as the oncogenes Calcium Voltage-Gated Channel Subunit Alpha1 D (*CACNA1D*), KIT Proto-Oncogene, Receptor Tyrosine Kinase (*KIT*), Mitogen-Activated Protein Kinase Kinase 4 (*MAP2K4*) and *COL4A2*, emerged in interaction with BMI, but their implication in the BMI to CRC risk association are yet to be elucidated.^42^, ^43^, ^44^, ^45^ Important modulators of the anti-tumour immune response, such as Spleen Associated Tyrosine Kinase (*SYK*) and Vascular Endothelial Growth Factor A (*VEGFA*), and several other inflammatory and pro-inflammatory cytokine coding genes [such as interleukin (IL)-*9*, *IL12B,* CD40 Molecule (*CD40*), *VEGFA,* TNF Superfamily Member 10 (*TNFSF10*)], and related immune-regulatory paths, were highlighted for NSAIDs.^46^ NSAIDs can inhibit the activation of cancer pathways (NF-κB) related to increased proliferation and survival, independently of COX-2 inhibition (which is thought to be the primary mechanism via which NSAIDs exert their anti-inflammatory and anti-tumour effects).^47^, ^48^, ^49^ In addition, several oncogenes like MET Proto-Oncogene, Receptor Tyrosine Kinase (*MET*), Raf-1 Proto-Oncogene, Serine/Threonine Kinase (*RAF1*), MYC proto-oncogene, bHLH transcription factor (*MYC*) were enriched for NSAIDs.^50^^,^^51^ Many of the above genes and their gene products have been previously identified as potential therapeutic drug targets of high priority, and the results of the present analysis provide additional evidence to support their association with CRC risk.^52^ Moreover, our analysis identified several other genes that are less studied in relation to the investigated exposures, such as argonaute RISC component, UDP-glucuronosyltransferase, and taste receptor coding genes, however their role in CRC tumorigenesis remains to be explained.

Overall, the results of the present analysis are characteristic of the breadth of mechanisms via which modifiable exposures, like excess body weight and smoking, act to increase CRC risk.^53^ Several key molecular pathways, that fall under the *sustaining proliferative signalling* and *evading growth suppressors* hallmark capabilities, such as the PI3K-Akt and MAPK signalling pathway, signalling by GPCR and Receptor Tyrosine Kinases (RTK), were enriched in the analysis for BMI and smoking.^54^^,^^55^ Pre-clinical models have shown that increased adiposity may drive epithelial mesenchymal transition (EMT), while various groups of active compounds in cigarette smoke can induce EMT via different signalling pathways.^56^^,^^57^ Several genes [Notch Receptor 2 (*NOTCH2*), mitogen-activated protein kinase 1 (*MAPK1*), Collagen Type IV Alpha 1 Chain (*COL4A1*)] and pathways that are involved in EMT, such as the Notch, TGF-beta, Wnt–β-catenin, Hedgehog, RTK, were also enriched for BMI and smoking.^56^ Obesity has been associated with cell structure changes that render the tissue microenvironment more favourable for tumour growth and metastasis.^58^ Genes such as Collagen Type IV Alpha 3 Chain (*COL4A3*), platelet derived growth factor receptor alpha (*PDGFRA*), syndecan 1 (*SDC1*), and pertinent pathways potentially implicated in the process of tissue remodelling were enriched for BMI.^59^ Additionally, increased adiposity is a condition characterized by metabolic dysfunction that results in *deregulation of cellular energetics*, another hallmark of cancer.^60^ Obesity affects the secretion of various endogenous hormones, like insulin and Insulin Like Growth Factor 1 (*IGF1*), estrogens, leptin and other adipokines, that apart from the effect they have on downstream proliferation-promoting cascades, also affect metabolism-related pathways.^61^ Several pathways directly related to metabolic reactions (metabolism of lipids, carbohydrates, glycolysis and gluconeogenesis), as well as hormone receptor coding genes, such as the Leptin Receptor (*LEPR)*, insulin receptor (*INSR*), estrogen receptor 1 (*ESR1*), and hormone-associated metabolic pathways were enriched for BMI.

Concomitantly enriched pathways and genes for different exposures were highlighted in our study. There was a notable overlap of enriched genes and pathways between BMI and NSAIDs, which suggests that potentially the BMI associated pathways may be particularly relevant to the aspirin/NSAID related protective association. Apart from key cancer-associated genes and pathways, such as BCL2 like 1 (*BCL2L1*; via cytokine-mediated signalling), and the Jak/STAT and PI3K-Akt pathways, several immune-regulatory, hormone-related, as well as cell-structure and motility-associated pathways were enriched. Adipose tissue contains numerous immune cells that create an inflammatory microenvironment, and several experimental studies have demonstrated a link between obesity and immunoregulatory proteins [such as programmed cell death 1 ligand 2 (*PDCD1LG2*)].^62^ Such adipose tissue derived inflammation contributes to insulin resistance, while inflammatory proteins may interact with adipokines and estrogens.^63^, ^64^, ^65^ Interestingly, hormone receptors like *INSR*, *LEPR*, *ESR1* were concomitantly enriched for BMI and NSAIDs. *SDC1,* a key cell surface adhesion molecule, that plays an important role in maintaining cell morphology, was also commonly enriched for those two exposures.^66^ Previous observational studies have shown a potential interaction of smoking and BMI in the NSAID-CRC association.^67^^,^^68^ The protective effect of aspirin on CRC was stronger among never-smokers compared to smokers, and the association between any NSAID use and CRC risk was attenuated with increasing BMI.^68^, ^69^, ^70^ Bitter taste receptor coding genes, such as Taste 2 Receptor Member 14 (*TAS2R14*), and Taste 2 Receptor Member 46 (*TAS2R46*) were highlighted in interaction with both BMI and calcium. Emerging evidence suggests that bitter taste receptors may play a role in modulating early tumorigenic processes in colorectal cancer.^71^ Potentially important commonalities observed for BMI and smoking include nuclear receptor corepressor 2 (*NCOR2*), a gene that encodes for a nuclear receptor co-repressor, which mediates transcriptional silencing of various genes, and *PDGFRA*, a gene that encodes for a cell surface tyrosine kinase receptor of platelet-derived growth factors.^72^^,^^73^ Both these genes have been characterized as promising therapeutic targets for CRC as they have received high OTP overall association scores (>0.5). Recent studies have linked both obesity and smoking with epigenetic alterations, and differential mRNA expression of *NCOR2,* but evidence for a common mechanism for *PDGFRA* is limited.^74^^,^^75^ Few commonalities were observed between BMI and physical activity. Notable ones include BIOCARTA's circadian pathway, identified using the ADABF approach, as well as pathways related to extracellular matrix organization, identified using ORA. An interesting commonality for NSAIDs and physical activity via the Basic Helix-Loop-Helix ARNT Like 1 (*BMAL1/ARNTL1)* gene, a transcriptional activator forming a core component of the circadian clock, was also observed. Regular physical activity positively influences circadian rhythmicity, and NSAID use has also been shown to affect the expression of circadian clock genes, whereas circadian disruption has been associated with increased risk of CRC.^76^, ^77^, ^78^ STAT protein coding genes [signal transducer and activator of transcription 3 (*STAT3*), signal transducer and activator of transcription 5B (*STAT5B*)], and related pathways, such as the IL9 and IL21 signalling pathways, that mediate cellular responses to interferons, and likely involved in the *Sustained proliferative signalling* hallmark, were enriched for calcium and smoking. In a potentially similar context, IFN gamma signalling pathway [and the Janus kinase 1 (*JAK1*) gene], was enriched for both calcium and fibre. Poly(ADP-ribose) polymerase 1 (*PARP1*), a gene that mediates significant post-translational modification of proteins that plays a key role in DNA repair, falling under the *Enabling Replicative Immortality*, and *Genome Instability and Mutation* hallmarks was enriched for both Alcohol and NSAIDs, potentially suggesting a common mechanism between the two exposures. We found a processed meat with fruit intake commonality, of the Argonaute protein coding genes Argonaute RISC Component 1 (*AGO1*) and Argonaute RISC Component 3 (*AGO3*), within the Dicer pathway, that are used for RNA-mediated gene silencing. Several genes from the UDP-glucuronosyltransferase family, including UDP Glucuronosyltransferase Family 1 Member A4 (*UGT1A4*), were identified in relation to both red meat intake and smoking, potentially indicating shared mechanisms involving xenobiotic metabolism.^79^ However, there is limited evidence in the current literature to support these as common underlying pathways for CRC risk. Further research is needed to determine whether the observed overlaps reflect true shared biological mechanisms. Gaining a deeper understanding of these potential commonalities could help refine cancer prevention guidelines and support the development of more targeted preventive strategies, particularly for individuals at higher CRC risk.

Although we used a relatively relaxed threshold for SNPs mapping to genes as being significant in the ORA analysis, there still might be significant loss of information due to the imposed *P*-value cutoff, and genes with marginally significant signals may have been ignored. This is a known limitation of ORA. On the other hand, the ADABF incorporates continuous measures of evidence strength (betas and corresponding variances from the interaction models), which is an advantage of association analysis. It is also well known that a method testing the association null hypothesis has a larger power compared to ORA which is an enrichment analysis method. This means that the ADABF method is more likely to reject the null hypothesis for more pathways than an enrichment method. These method characteristics can explain the differences in the results between the ORA and ADABF approaches, and the fact that more genes and pathways were found to be associated with exposures with stronger effects with CRC risk, like BMI, and smoking. Although pathways identified by both methods can be considered robustly enriched and prioritized for CRC risk, those identified by only one method should not be disregarded. Instead, they should be explored complementarily in future investigations. Even though we restricted our analysis to individuals of European ancestry and adjusted for three principal components to capture remaining variability, subtle population structure may still confound GWAS results, particularly for traits influenced by environmental or lifestyle factors. Such residual stratification could bias genetic associations and potentially lead to spurious findings.

The large sample size, and the high-quality phenotype and genotype data may minimize potential sources of bias and are among the strengths of our study. A wide range of established CRC risk factors were included, and several gene-set libraries were used to cover a variety of biological pathways, in the pathway enrichment analysis. However, exposure assessments were self-reported in most of the studies included, measured at a single time point, and may have been subject to measurement errors. Additionally, the estimates on which the analysis was based were derived exclusively from European ancestry populations, which limits generalizability. To maximize statistical power, our analysis focused on CRC overall, without performing stratified analyses by subsite (e.g., right colon, left colon, and rectum), sex, or early-onset CRC. Given that associations with CRC risk may vary across these subgroups and considering the shifting prevalence of CRC risk factors in populations over time, future studies should explore these associations within specific strata.

The results of the present analysis may aid in elucidating the etiological associations of risk factors to CRC risk and inform personalized prevention strategies.

## Contributors

EB: Data curation, Formal analysis, Methodology, Investigation, Visualization, Writing–Original Draft, Writing–Review & Editing; RY: Data verification, Data curation, Formal analysis, Writing–Review & Editing; AEK: Data verification, Data curation, Formal analysis, Writing–Review & Editing; GM, NM, DA, LNA, ELB, SIB, TDB, HB, ABH, PTC, RCT, ATC, IC, MAD, VDO, ND, DAD, SBG, AG, MH, LH, JRH, EK, TOK, AK, SK, LLM, JPL, LL, BML, VM, JM, JM, CCN, MOS, JRP, NP, AJP, ARP, PDPP, EAP, CQ, ERN, JSM, RES, MCS, CET, YT, CYM, KV, PV, VV, EW, AW, MOW, AHW, MJG: Methodology, Writing–Review & Editing; WJG, UP, ME: Conceptualization, Investigation, Methodology, Writing–Review & Editing, Supervision; KKT: Conceptualization, Funding acquisition, Investigation, Methodology, Writing–Review & Editing, Supervision. All authors critically revised the manuscript for intellectual content. All authors read and approved the final manuscript.

## Data sharing statement

Data described in the manuscript will be made available upon request pending application and approval to the GECCO consortium.

## Declaration of interests

EB was supported by grants from Cancer Research UK (CRUK; grants number: PPRCPJT∖100005), and World Cancer Research Fund International (WCRF; IIG_FULL_2020_022). RY was supported by Cancer Research UK (CRUK; grants number: PPRCPJT∖100005). ELB was supported by the National Cancer Institute, National Institutes of Health. SB was supported by the Intramural Research Program of the Division of Cancer Epidemiology and Genetics, National Cancer Institute, NIH. ATC was supported by grants from the National Institutes of Health, Stand Up to Cancer, Freenome Holdings, and American Cancer Society; Received payment for expert testimony by Boehringer Ingelheim; Participation on a Data Safety Monitoring Board or Advisory Board for Pfizer Inc; Held leadership or fiduciary role in the American Gastroenterological Association, and National Cancer Institute. DAD was supported by a National Institutes of Health grant. ESK was supported by grants from the National Cancer Institute [R01 CA201407, P01 CA196569]; Holds stocks or stock options from Abbvie (ABBV), and Pfizer (PFE). TOK was supported by a National Cancer Institute grant [R01CA066635, 1996–2008]. AK was supported by a National Institutes of Health grant [R01CA273198]; Was a consultant with Bristol Myers Squibb, Inari Agriculture, and Arcadia Science; Holds stocks or stock options from TensorBio Inc, SerImmune Inc., Illumina, Deep Genomics, ImmunAI. BML was a consultant with Elsevier; Was supported for attending meetings and/or travel by the International Epidemiological Association; Held leadership or fiduciary role in the International Epidemiological Association Council. VM was supported by grants from the Instituto de Salud Carlos III, Programa FORTALECE del Ministerio de Ciencia e Innovación through the project number FORT23/00032, Spanish Association Against Cancer (AECC) Scientific Foundation grant GCTRA18022MORE, and Consortium for Biomedical Research in Epidemiology and Public Health (CIBERESP) action Genrisk. JLM was supported by grants from the National Cancer Institute [R01 CA201407, P01 CA196569, R01 CA273198]. JRP was supported by a National Institutes of Health grant. AJP Was a consultant with Abbvie. PDPP was supported by a Cancer Research UK grant. EAP held leadership or fiduciary role (unpaid), as an elected member of the American Association for Cancer Research Board of Directors. RES was supported by grants from the National Cancer Institute NCI EDRN Grant (U01-CA152753), and Exact Sciences, Freenome, Immunovia; Was a consultant with Guardant, Freenome, Exact Sciences; Participated on a Data Safety Monitoring Board or Advisory Board in VA CONFIRM trial, NCI Cancer Screening Research Network. CET is an epidemiology contractor with Pfizer, unrelated to the present study. VV was supported by a Czech Science Foundation 21-04607X grant, unrelated to the submitted work. AW was supported by a Swedish Cancer Foundation grant. WJG was supported by grants from the National Cancer Institute [R01 CA201407, P01 CA196569, R01 CA273198]. UP was supported by grants from the National Institute of Health, V Foundation, and Goldman Sachs Foundation; Was a consultant with AbbVie; UP's family is holding individual stocks for the following companies: Amazon, ARM Holdings PLC, BioNTech, BYD Company Limited, Crowdstrike Holdings Inc, CureVac, Google/Alphabet, Microsoft Corp, NVIDIA Corp, Stellantis. ME was supported by Cancer Research UK (CRUK; grants number: PPRCPJT∖100005). KKT was supported by grants from Cancer Research UK (CRUK; grants number: PPRCPJT∖100005), and World Cancer Research Fund International (WCRF; IIG_FULL_2020_022).The remaining authors have no competing interests to declare.
