# Supplementary material for: Using gene-environment interactions to explore pathways for colorectal cancer risk
Source: eBioMedicine. 2025 Oct 11;121:105964. doi: 10.1016/j.ebiom.2025.105964 (PMC12547926; doi:10.1016/j.ebiom.2025.105964)
Supplement: Methods Supplement [file mmc1.docx]

**Methods supplement**

We used a total of 2950 gene-sets of the Canonical pathways (CP) collection of the Human Molecular Signatures Database (MSigDB) **[1]**. Gene sets in this collection have been curated from various sources, including pertinent online pathway databases and biomedical literature. Many sets are also contributed by individual domain experts. Each of these databases offers unique features and provides a broad and complementary array of pathway resources.

The pathway gene sets are curated from the following online databases:

**KEGG (legacy sets,** [**http://www.pathway.jp**](http://www.pathway.jp/)**) [2].**

The Kyoto Encyclopedia of Genes and Genomes (KEGG) is a comprehensive, curated database that provides information on biological systems, including cellular processes, biochemical pathways, human diseases, drugs, and gene functions. Developed by Professor Minoru Kanehisa in Japan in 1995, KEGG is widely used in genomics, bioinformatics, and molecular biology to study biological interactions and interpret high-throughput experimental data, such as gene expression and proteomics.

The KEGG Pathway database is a central part of the Kyoto Encyclopedia of Genes and Genomes (KEGG), offering a rich resource for visualizing and understanding molecular interactions and processes within cells. This part of KEGG focuses on pathway maps that represent various biological processes, including metabolic pathways, signaling cascades, gene regulatory networks, and disease-related pathways. Each pathway map in KEGG Pathways is organized to show the flow of biochemical reactions and gene-product interactions, typically organized hierarchically from simple biochemical interactions to complex physiological processes.

KEGG provides extensive maps of metabolic processes, encompassing cellular respiration, photosynthesis, lipid biosynthesis, amino acid metabolism, and others. KEGG also covers signaling pathways, such as those involved in cell communication, immune response, and cancer pathways. Signaling maps often include details on how receptor proteins, secondary messengers, and transcription factors work together to influence gene expression and cellular functions. A unique feature of KEGG Pathways is its representation of disease-related pathways, particularly those relevant to human health, such as cancer, metabolic disorders, and neurodegenerative diseases. These pathways integrate known genetic, molecular, and environmental factors contributing to disease mechanisms.

At the time of assessment, there were 186 KEGG pathways in the MSigDB resources, including a total of 4,824 unique genes, with a mean of 64.2 genes per pathway (range: 7-379 genes).

**BIOCARTA [3].**

The BIOCARTA database is a resource focused on gene sets associated with a variety of molecular and cellular processes. Originally developed as a collection of curated biological pathways, BIOCARTA offers a set of pathway maps illustrating cellular and molecular functions, emphasizing key signaling pathways, cellular structural pathways, and metabolic processes. BIOCARTA is no longer updated independently, but It is maintained within bioinformatics tools, such as Gene Set Enrichment Analysis (GSEA) and the MSigDB, BIOCARTA remains a valuable resource for understanding biological interactions at the pathway level.

BIOCARTA’s pathways notably emphasize signal transduction networks, which are essential for understanding how cells respond to external stimuli like growth factors, cytokines, and environmental stressors. These pathways help elucidate processes by which cells communicate and regulate functions such as proliferation, differentiation, and apoptosis. BIOCARTA includes pathways that illustrate mechanisms of cell cycle control and apoptosis (programmed cell death), crucial in understanding cancer and other diseases involving unchecked cell growth. By representing the interplay of oncogenes, tumor suppressors, and other regulatory proteins, BIOCARTA’s pathways aid in researching cell cycle dysregulation and programmed cell death. BIOCARTA pathways also encompass several metabolic processes, providing insight into the biochemical networks that drive cellular metabolism, such as ATP generation, amino acid biosynthesis, and lipid metabolism. These pathways are foundational for studying how metabolic imbalances may contribute to conditions like diabetes, obesity, and cancer. Pathways in BIOCARTA address cellular structure by illustrating the role of cytoskeletal components, such as actin and microtubules, in maintaining cell shape, motility, and intracellular transport. These pathways are essential for understanding diseases where cellular architecture is disrupted.

At the time of assessment, there were 292 BIOCARTA pathways in the MSigDB resources, including a total of 1,432 unique genes, with a mean of 15.8 genes per pathway (range: 4-78 genes).

The National Cancer Institute **Pathway Interaction Database (PID)** (<http://pid.nci.nih.gov>) **[4].**

The Pathway Interaction Database (PID) was a collaborative effort between the National Cancer Institute (NCI) and the Nature Publishing Group, designed to provide a comprehensive catalog of curated pathways and molecular interactions, especially those relevant to cancer research. This resource, although archived since 2013, still serves as a valuable reference in bioinformatics for its detailed representation of protein-protein interactions, cell signaling pathways, and transcriptional regulatory pathways. PID's pathways cover several core processes, including cell cycle regulation, apoptosis, immune response, and signal transduction. Each pathway entry includes detailed diagrams, references to primary literature, and descriptions of molecular interactions and functions. The PID is now integrated into other databases, but its standardized, expert-reviewed information remains widely referenced in tools like Cytoscape and BioPAX to analyze network data in cancer and other diseases.

At the time of assessment, there were 196 PID pathways in the MSigDB resources, including a total of 2,384 unique genes, with a mean of 39.5 genes per pathway (range: 9-133 genes).

**REACTOME (**[**http://www.reactome.org**](http://www.reactome.org/)**) [5].**

REACTOME is an open-source, peer-reviewed pathway database, maintained by an international team from OICR, OHSU, EMBL-EBI, and NYULMC. Reactome's core unit is the reaction, with entities like nucleic acids, proteins, complexes, and small molecules participating in reactions to form a network of biological interactions. It is designed to represent the entire spectrum of human biological processes, include signal transduction, immune function, transcriptional regulation, programmed cell death, and intermediary metabolism. Each pathway in REACTOME is manually curated and supported by literature references, making it a reliable resource for biomedical research. Importantly, REACTOME pathways are computationally derived for comparative analyses across species, which is useful in translational research and drug discovery.

At the time of assessment, there were 1,614 REACTOME pathways in the MSigDB resources, including a total of 9,756 unique genes, with a mean of 48.8 genes per pathway (range: 3-1,287 genes).

**WikiPathways (**[**https://www.wikipathways.org/**](https://www.wikipathways.org/)**) [6].**

WikiPathways (WP) covers a wide range of biological processes, such as metabolic, signaling, and regulatory pathways. A unique aspect of WP is its collaborative model, which enables researchers to continuously update pathways with new findings, ensuring that the content remains current and reflects a broad range of expertise. The platform is integrated with Ensembl, UniProt, and other databases to provide detailed gene and protein annotations within pathways. WP also supports the analysis of omics data, as it can be easily incorporated into analysis tools for pathway enrichment studies.

At the time of assessment, there were 662 WP pathways in the MSigDB resources, including a total of 6,720 unique genes, with a mean of 41.6 genes per pathway (range: 2-420 genes).

**References**

[1] Liberzon A, Birger C, Thorvaldsdóttir H, Ghandi M, Mesirov JP, Tamayo P. The Molecular Signatures Database (MSigDB) hallmark gene set collection. Cell systems. 2015;1(6):417-25.

[2] Kanehisa M, Goto S. KEGG: kyoto encyclopedia of genes and genomes. Nucleic Acids Res. 2000;28(1):27-30.

[3] Nishimura, D. (2001). "BioCarta." Biotech Software & Internet Report 2(3): 117-120.

[4] Schaefer CF, Anthony K, Krupa S, Buchoff J, Day M, Hannay T, et al. PID: the Pathway Interaction Database. Nucleic Acids Res. 2009;37(Database issue):D674-9.

[5] Fabregat A, Sidiropoulos K, Viteri G, Forner O, Marin-Garcia P, Arnau V, et al. Reactome pathway analysis: a high-performance in-memory approach. BMC Bioinformatics. 2017;18(1):142.

[6] Slenter DN, Kutmon M, Hanspers K, Riutta A, Windsor J, Nunes N, et al. WikiPathways: a multifaceted pathway database bridging metabolomics to other omics research. Nucleic Acids Research. 2017;46(D1):D661-D7.
