# Supplementary material for: Using gene-environment interactions to explore pathways for colorectal cancer risk
Source: eBioMedicine. 2025 Oct 11;121:105964. doi: 10.1016/j.ebiom.2025.105964 (PMC12547926; doi:10.1016/j.ebiom.2025.105964)
Supplement: S Figure 1 [file mmc2.pdf]

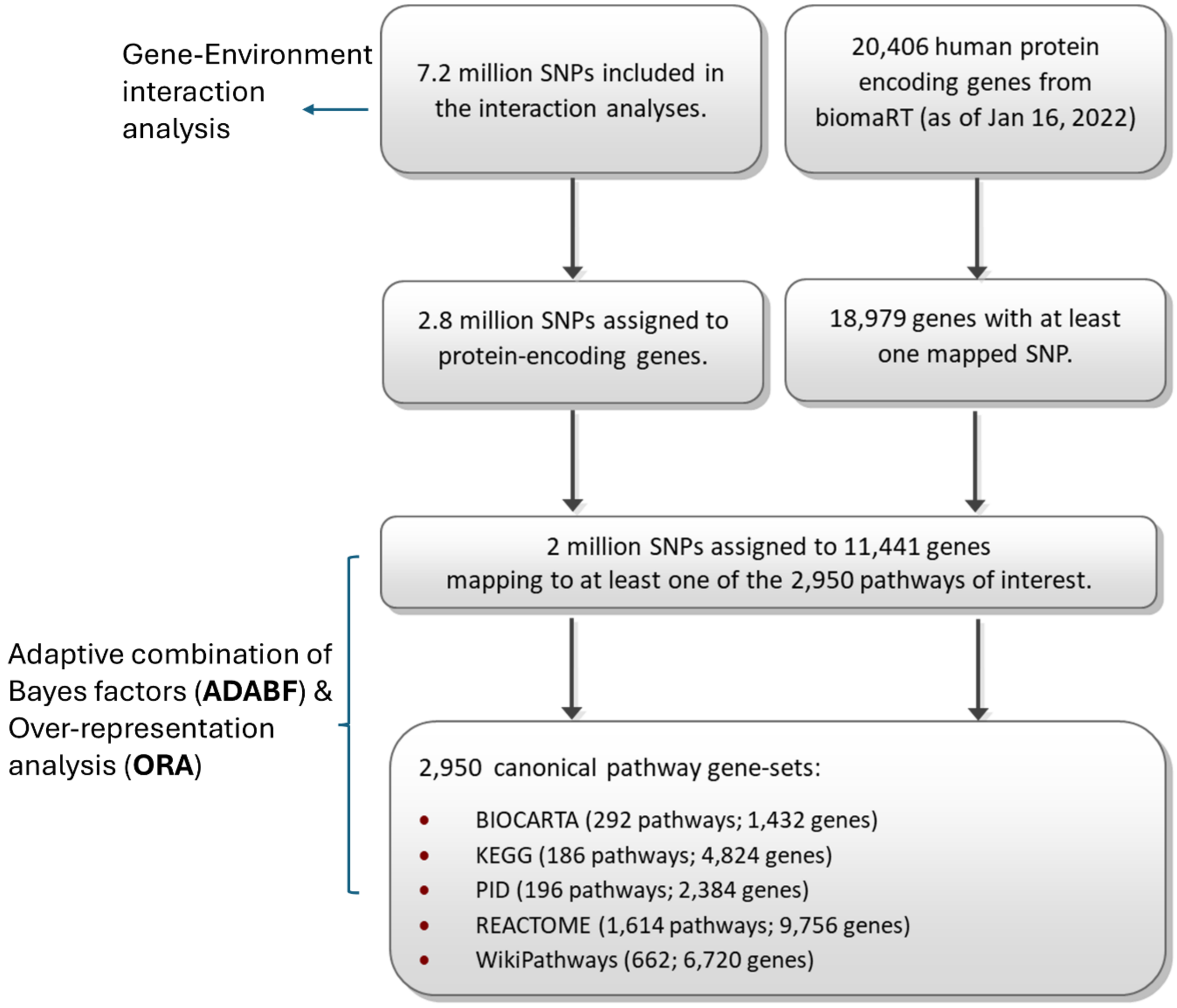

S Figure 1. Flow chart of the mapping of variants to genes and gene-sets (pathways).

KEGG: Kyoto Encyclopedia of Genes and Genomes; PID: Pathway Interaction Database; SNPs: Single nucleotide polymorphisms.
